# Associations between homocysteine, inflammatory cytokines and sarcopenia in Chinese older adults with type 2 diabetes

**DOI:** 10.1186/s12877-021-02622-y

**Published:** 2021-12-15

**Authors:** Zhi-Jing Mu, Jun-Ling Fu, Li-Na Sun, Piu Chan, Shuang-Ling Xiu

**Affiliations:** 1 Department of Endocrinology, Beijing Institute of Geriatrics, Xuanwu Hospital, Capital Medical University, Beijing, 100053 China; 2 Department of Neurobiology, Neurology and Geriatrics, Xuanwu Hospital of Capital Medical University, Beijing Institute of Geriatrics, Beijing, 100053 China; 3 Clinical Center for Parkinson’s Disease, Capital Medical University, Beijing, China; 4 Key Laboratory for Neurodegenerative Disease of the Ministry of Education, Beijing Key Laboratory for Parkinson’s Disease, Parkinson Disease Center of Beijing Institute for Brain Disorders, Beijing, China; 5 National Clinical Research Center for Geriatric Disorders, Beijing, China; 6 Advanced Innovation Center for Human Brain Protection, Capital Medical University, Beijing, China

**Keywords:** Homocysteine, Inflammatory cytokines, Sarcopenia, Type 2 diabetes, Older adult

## Abstract

**Background:**

Sarcopenia, an age-related disease, has been implicated as both a cause and consequence of type 2 diabetes mellitus (T2DM) and a symbol of poor prognosis in older adults with T2DM. Therefore, early detection and effective treatment of sarcopenia are particularly important in older adults with T2DM. We aimed to investigate the prevalence of sarcopenia in Chinese older T2DM patients and explore whether homocysteine and inflammatory indexes could serve as biomarkers and participate in the development process of sarcopenia.

**Methods:**

T2DM patients aged over 60 years were consecutively recruited from the ward of department of Endocrinology, Xuanwu Hospital between April 2017 and April 2019. Sarcopenia was defined based on the standard of the Asian Working Group of Sarcopenia, including muscle mass, grip strength and gait speed. Logistic regression was used to explore the association between biochemical indicators and sarcopenia. Receiver operating characteristic (ROC) curves were applied to determine the diagnostic effect of these clinical indicators.

**Results:**

Totally 582 older adults with T2DM were characterized and analyzed in the study. Approximately 8.9% of the older T2DM patients had sarcopenia. After adjusting for age, sex, body mass index (BMI) and hemoglobin A1c (HbA1c), increased concentrations of homocysteine [odds ratio (OR): 2.829; 95% confidence interval (CI), 1.064–7.525] and high-sensitive C-reactive protein (hs-CRP) (OR: 1.021; 95% CI, 1.001–1.042) were independent predictors of sarcopenia; but not interleukin-6. The combination of age, sex, BMI and HbA1c provided a discriminatory effect of sarcopenia with an area under the curve (AUC) of 0.856, when homocysteine was added to the model, the value of the ROC curve was further improved, with an AUC of 0.861.

**Conclusion:**

In the current study, we demonstrated a positive correlation of homocysteine, hs-CRP with sarcopenia in older adults with T2DM and the relationship remained significant even after adjustment for HbA1c. These biomarkers (homocysteine and hs-CRP) may play important roles in the pathological process of sarcopenia.

## Background

Sarcopenia was defined as a muscle disease rooted in adverse muscle changes that accrue across a lifetime by The European Working Group on Sarcopenia in Older People 2 (EWGSOP2) in 2019 [1], which means it is becoming a global health problem. Sarcopenia is characterized by decreased muscle strength and physical conditions, which lead to an increased risk of falls, fractures and mortality [2].

The prevalence of sarcopenia varies widely among studies because of the controversy definition of sarcopenia [3]. A study of community dwelling older adults (average age of 67 years) in UK found the prevalence of sarcopenia was 4.6% in men and 7.9% in women according to EWGSOP definition [4]. In a community-dwelling elderly Japanese population, the prevalence of sarcopenia ranged from 2.5 to 98.0% in men and 2.3 to 88.0% in women based on different sarcopenia definitions [5]. In Turkey, sarcopenia was diagnosed in 73.3% of the nursing home residents [6], while the prevalence of sarcopenia defined by EWGSOP was 0.8% (1.3% in men and 0.6% in women) in community-dwelling outpatient older adults [7]. Other studies also reported the prevalence of sarcopenia differed among different populations, ages, sex as well as living settings (community or nursing homes) [8–10].

Epidemiological evidence has shown that the prevalence of diabetes is 12.8% among adults living in China and the prevalence of diabetes is even higher among adults aged 50 [11]. Numerous studies have reported that the possibility of sarcopenia is much higher in type 2 diabetes mellitus (T2DM) [12, 13]. Wang et al. demonstrated that the prevalence of sarcopenia was significantly higher in patients with diabetes than in healthy subjects (14.8% vs. 11.2%), the patients with T2DM were 1.56-fold likely to exhibit sarcopenia compared to healthy controls [14].

Reportedly, sarcopenia is associated with a 1.5–2-fold increased risk of falls and fractures, especially in older adults [15], and diabetes is also correlated with a higher risk of fractures independently of bone mineral density [16]. Thus, sarcopenia may be associated with an increased risk of diabetes-related fractures [17] and it is crucial to identify subjects with sarcopenia in patients with T2DM. Importantly, despite the attention given to the diagnosis of sarcopenia, poor management is still performed, mainly related to physical therapy [18]. Searching for key biomarkers can assist with further comprehension of the pathophysiological disease processes, which can help with the early detection of sarcopenia and the selection of the optimal pharmacologic agents for interventions. Previous experimental data have suggested that the pathogenesis of sarcopenia might be attributed to decreases in hormone levels, neuromuscular dysfunction and inflammation [19]. Chronic inflammation is considered as a hazardous factor for sarcopenia [19]. Visser et al. reported a negative association of inflammatory indicators with handgrip strength [20]. A meta-analysis compared inflammatory markers in participants with and without sarcopenia, and found that high-sensitivity C-reactive protein (hs-CRP) demonstrated a significantly positive correlation with sarcopenia [19]. Furthermore, homocysteine, an indicator closely related to inflammation, plays an important role in many age-correlated diseases, including cardiovascular diseases and sarcopenia [21]. Whether these correlations could apply to older Chinese adults with T2DM or nor, and what’s the pathophysiological roles that hs-CRP and homocysteine play in the development of sarcopenia remain unknown. In the present study, based on a well-characterized T2DM cohort, we aimed to explore the prevalence of sarcopenia and examined the associations of homocysteine and inflammatory cytokines with sarcopenia and its components.

## Methods

### Study participants

Older adults with T2DM aged over 60 years were consecutively recruited from the ward of the department of Endocrinology, Xuanwu Hospital, Capital medical university between April 2017 and April 2019. The inclusion criteria were Chinese who met the criteria of the American Diabetes Association for T2DM [22]. The exclusion criteria were: 1) the presence of a currently progressive illness (fever, infection, acute cerebrovascular disease, sepsis, acute coronary syndrome, gastrointestinal bleeding, acute liver failure, acute respiratory failure, anemia, etc.); 2) inability to complete the 6-m walking speed and grip strength measurement (mainly to exclude patients who have neurological disease or sequelae of cerebrovascular disease); 3) type 1 diabetes mellitus; diabetic ketoacidosis; 4) have serious bone and joint disease or neuromuscular disease that affect the daily activities. Totally 631 inpatients were recruited, among which, 49 of them were excluded from this study. Among these 49 subjects, 21 refused to participate in the study, 14 subjects had acute problems, 8 participants were unable to cooperate with inspection due to disability and 6 had missing data. Consequently, a total of 582 participants were analyzed in the current study. The study was approved by Xuanwu Hospital of Capital Medical University, China (approval number: CTR-IPR-2019002). All subjects provided informed consent for the study.

### Clinical and biochemical measurements

Clinical indexes including the height and weight of each individual were measured. Body mass index (BMI) was calculated as weight (kg)/height(m)^2^. Blood samples were obtained after a 10-h overnight fast. Fasting glucose, lipids, alanine aminotransferase, aspartate aminotransferase, uric acid, creatinine, homocysteine and fasting C-peptide were measured by an automatic biochemical analyser (BioTek Instrument, Inc., Beijing, China). Hemoglobin A1c (HbA1c) was determined by high-performance liquid chromatography. Complete blood count was assessed. Serum levels of hs-CRP were assessed by an immunoturbidimetry assay (Kanto Chemical Co Inc., Tokyo, Japan). Interleukin-6 (IL-6) was assayed using an enzyme-linked immunosorbent assay kit (Beijing Biolab Science and Technology Co. Ltd., Beijing, China). We assessed the incidence of diabetic complications, including diabetic nephropathy, diabetic retinopathy, and diabetic peripheral neuropathy.

### Sarcopenia and related measurements

Sarcopenia was defined according to the recommendation from the Asian Working Group for Sarcopenia (AWGS) [23], as the presence of low muscle mass with low muscle strength or/and low physical performance. Handgrip strength was assessed by the Jamar® Hydraulic Hand Dynamometer (Patterson Medical, Warrenville, IL, USA). Each side was measured three times and the maximal grip strength was selected for the analyses. Low grip strength was considered < 26 kg for men and < 18 kg for women [23]. Gait speed for a 6-m distance was measured by a stopwatch. Two timed trials were taken altogether and the fastest pace was used for the analysis. Low gait speed was considered < 0.8 m/s [23]. Appendicular skeletal muscle mass (SMI) was calculated as appendicular skeletal muscle (ASM)/height^2^ (kg/m^2^). ASM was determine by dual-energy X-ray absorptiometry (DXA, LUNAR iDXA, USA) and was calculated as the sum of arm and leg skeletal muscle mass. A single experienced technologist performed all the scans. Low muscle mass was considered SMI < 7.0 kg/m^2^ for men, SMI < 5.4 kg/m^2^ for women [23]. The measurements of handgrip strength and gait speed were performed by two trained and experienced nurses.

### Statistical analysis

All statistical analyses for the present study were performed using Statistical Package for Social Science (SPSS) version 24.0. We performed normality analysis prior to analysis and skewed distributions were natural logarithmically transformed. The data are expressed as the mean ± standard deviation for continuous variables, and counts (percentages) for categorical variables. Student’s t test and chi-square tests were used for continuous and categorical variables, respectively. The associations of inflammatory cytokines with components of sarcopenia were analyzed via partial correlation coefficients. The variables detected as significant in univariate analyses were analyzed with logistic regression analyses, including age, sex, BMI and HbA1c. The potential risk factors for sarcopenia were selected for multi-factor receiver operating characteristic (ROC) analysis, we combined the selected indicators by regression model. First, perform logistic regression analysis on the selected variables to find the predicted probability value as a new variable. Subsequently, ROC curves were used to evaluate the ability of the predicted probability value for diagnosing sarcopenia. Logistic regression was utilized to explore the correlations between sarcopenia and serum homocysteine, hs-CRP and IL-6 and the results are presented as odds ratios (ORs) and 95% confidence intervals (CIs). A *P* value < 0.05 and 95% CI not crossing the null value were considered statistically significant.

## Results

### Clinical features and inflammatory factors of sarcopenia

The basic characteristics of the study subjects are summarized in Table 1. Of all the 582 T2DM subjects, 52 (8.9%) met the diagnostic criteria of sarcopenia. Compared to the non-sarcopenic group, the sarcopenic patients were older (74.0 ± 7.8 y vs. 67.0 ± 6.6 y, *P* <  0.001), had lower BMI (22.9 ± 3.4 kg/m^2^ vs. 26.0 ± 3.5 kg/m^2^, *P <* 0.001) and higher HbA1c (9.2 ± 2.2% vs. 8.4 ± 1.8%, *P* = 0.008), while the two groups showed comparable lipids, diabetic complications, complete blood count, liver and kidney function. As expected, patients with sarcopenia demonstrated slower walking speed (0.8 ± 0.2 m/s vs. 1.1 ± 0.3 m/s, *P* <  0.001), lower SMI (5.9 ± 0.8 kg/m^2^ vs. 7.1 ± 1.0 kg/m^2^, *P* <  0.001) and decreased grip strength (23.0 ± 8.1 kg vs. 29.6 ± 10.1 kg, *P* <  0.001). With respect to inflammatory cytokines, subjects with sarcopenia had higher serum levels of ln-homocysteine (2.7 ± 0.5 umol/L vs. 2.5 ± 0.3 umol/L, *P* <  0.001) and hs-CRP (7.7 ± 18.0 mg/L vs. 4.2 ± 9.1 mg/L, *P* = 0.019) than controls but similar in serum ln-IL-6 concentrations (1.4 ± 0.8 ng/L vs. 1.6 ± 0.8 ng/L, *P* = 0.194).

**Table 1:** General characteristics of study subjects

	Non-sarcopenia	Sarcopenia	***P***
	***n*** = 530	***n*** = 52	
Age (y)	67.03 ± 6.60	74.04 ± 7.79	**< 0.001**
Sex (M/F)	255/275 (48%/52%)	36/16 (69%/31%)	**0.004**
Body mass index (kg/m^2^)	25.98 ± 3.51	22.90 ± 3.37	**< 0.001**
Diabetic peripheral neuropathy (Y/N)	247/283 (47%/53%)	23/29 (44%/56%)	0.880
Diabetic nephropathy (Y/N)	89/441 (17%/83%)	10/42 (19%/81%)	0.226
Diabetic retinopathy (Y/N)	87/443 (16%/84%)	13/39 (25%/75%)	0.755
White blood cell (× 10^9^/L)	6.26 ± 1.75	6.28 ± 1.45	0.936
Red blood cell (× 10^12^/L)	4.42 ± 0.53	4.18 ± 0.63	0.082
Platelets (×10^9^/L)	216.48 ± 60.78	195.42 ± 66.08	0.171
Hemoglobin (g/L)	134.95 ± 16.06	133.06 ± 16.15	0.422
Triglycerides (mmol/L)	0.42 ± 0.61	0.34 ± 0.51	0.324
Total cholesterol (mmol/L)	4.39 ± 1.12	4.34 ± 1.07	0.740
HDL-C (mmol/L)	1.21 ± 0.36	1.21 ± 0.46	0.940
LDL-C (mmol/L)	2.65 ± 0.92	2.59 ± 0.84	0.643
Uric acida (mmol/L)	5.73 ± 0.27	5.69 ± 0.34	0.348
Creatinine (μmoI/L)	69.00 ± 24.35	72.02 ± 30.83	0.407
Alanine aminotransferasea (U/L)	2.96 ± 0.51	2.89 ± 0.66	0.338
Aspartate aminotransferasea (U/L)	3.09 ± 0.32	3.11 ± 0.51	0.662
Fasting glucose (mmol/L)	9.31 ± 3.57	8.96 ± 3.55	0.501
Fasting C-peptide (ng/ml)	2.54 ± 1.51	2.65 ± 1.78	0.645
Hemoglobin A1c (%)	8.43 ± 1.83	9.16 ± 2.17	**0.008**
Homocysteine^a^ (umol/L)	2.52 ± 0.31	2.69 ± 0.45	**< 0.001**
Hs-CRP (mg/L)	4.15 ± 9.12	7.68 ± 17.97	**0.019**
Interleukin-6^a^ (ng/L)	1.40 ± 0.80	1.55 ± 0.81	0.194
ASM (kg)	19.52 ± 4.09	15.82 ± 3.22	**< 0.001**
SMI (kg/m^2^)	7.11 ± 1.02	5.85 ± 0.81	**< 0.001**
Grip strength (kg)	29.62 ± 10.05	23.02 ± 8.08	**< 0.001**
Walking speed (m/s)	1.10 ± 0.28	0.76 ± 0.23	**< 0.001**

Values in bold are significant at *P* < 0.05

^a^Skewed distributions were natural logarithmically transformed

Data were expressed as n (%), mean ± SD. *P* values are from Student’s t test or Chi-square tests

### Associations of homocysteine and inflammatory factors with components of sarcopenia

To explore the correlation of inflammatory cytokines and sarcopenia, we further utilized Pearson’s correlation to analyse the associations between homocysteine, inflammatory factors (hs-CRP, IL-6) and components of sarcopenia (SMI, grip strength and walking speed). As shown in Table 2, after adjusting for age, sex and BMI, homocysteine was negatively correlated with SMI (*r* = − 0.083, *P* = 0.047) and walking speed (*r* = − 0.204, *P* <  0.001). However, hs-CRP (*r* = − 0.113, *P* = 0.007) and IL-6 (*r* = − 0.086, *P* = 0.042) only demonstrated a negative association with SMI. No association was observed between inflammatory factors and grip strength. Notably, the above differences remained significant even after adjusting for HbA1c.

**Table 2:** Partial correlations between inflammatory markers, homocysteine and components of sarcopenia

	Homocysteine^**a**^		Hs-CRP		Interleukin-6^**a**^	
	*r*	*P*	*r*	*P*	*r*	*P*
**After adjusting for age, sex and BMI**						
SMI (kg/m^2^)	−0.083	**0.047**	−0.113	**0.007**	−0.086	**0.042**
Grip strength (kg)	−0.050	0.236	−0.047	0.259	−0.072	0.090
Walking speed (m/s)	−0.204	**< 0.001**	−0.048	0.256	−0.034	0.434
**After adjusting for age, sex, BMI and HbA1c**						
SMI (kg/m^2^)	−0.082	**0.050**	−0.107	**0.011**	−0.085	**0.046**
Grip strength (kg)	−0.050	0.238	−0.047	0.261	−0.072	0.091
Walking speed (m/s)	−0.204	**< 0.001**	−0.049	0.249	−0.034	0.432

Values in bold are significant at *P* < 0.05

r: Partial Correlation Coefficients

^a^Skewed distributions were natural logarithmically transformed

### Homocysteine and inflammatory factors as the independent predictor of sarcopenia

Separate modeled logistic regression was applied to assess each inflammatory marker as a predictor of sarcopenia. After adjustment for age, sex and BMI, increased concentrations of homocysteine (OR: 2.844; 95% CI, 1.071–7.553) and hs-CRP (OR: 1.021; 95% CI, 1.000–1.042) were independent predictors of sarcopenia but not IL-6 (Table 3). When all significant inflammatory factors were analyzed together, increased levels of homocysteine (OR: 2.809; 95% CI, 1.046–7.538) remained significantly correlated with sarcopenia. For every additional natural logarithm of homocysteine, the patients were 3 times more likely to have sarcopenia. Because the participants included in this study were all T2DM subjects, we further corrected HbA1c in the model, the predictive effect of homocysteine (OR: 2.829; 95% CI, 1.064–7.525) and hs-CRP (OR: 1.021; 95% CI, 1.001–1.042) were unaltered.

**Table 3:** Odds ratio for sarcopenia according to inflammatory markers and homocysteine

	OR (95%CI)	***P***
**After adjusting for age, sex and BMI**		
inflammatory markers modeled separately		
Homocysteine^a^	2.844 (1.071–7.553)	**0.036**
Hs-CRP	1.021 (1.000–1.042)	**0.046**
Interleukin-6^a^	1.245 (0.825–1.877)	0.297
inflammatory markers modeled together		
Homocysteine^a^	2.809 (1.046–7.538)	**0.040**
Hs-CRP	1.019 (0.998–1.041)	0.069
Interleukin-6^a^	1.195 (0.780–1.831)	0.412
**After adjusting for age, sex, BMI and HbA1c**		
inflammatory markers modeled separately		
Homocysteine^a^	2.829 (1.064–7.525)	**0.037**
Hs-CRP	1.021 (1.001–1.042)	**0.044**
Interleukin-6^a^	1.218 (0.804–1.846)	0.351
inflammatory markers modeled together		
Homocysteine^a^	2.820 (1.048–7.590)	**0.040**
Hs-CRP	1.019 (0.999–1.041)	0.066
Interleukin-6^a^	1.166 (0.756–1.797)	0.487

Values in bold are significant at *P* < 0.05

^a^Skewed distributions were natural logarithmically transformed

### ROC curve analysis

**Fig. 1:**
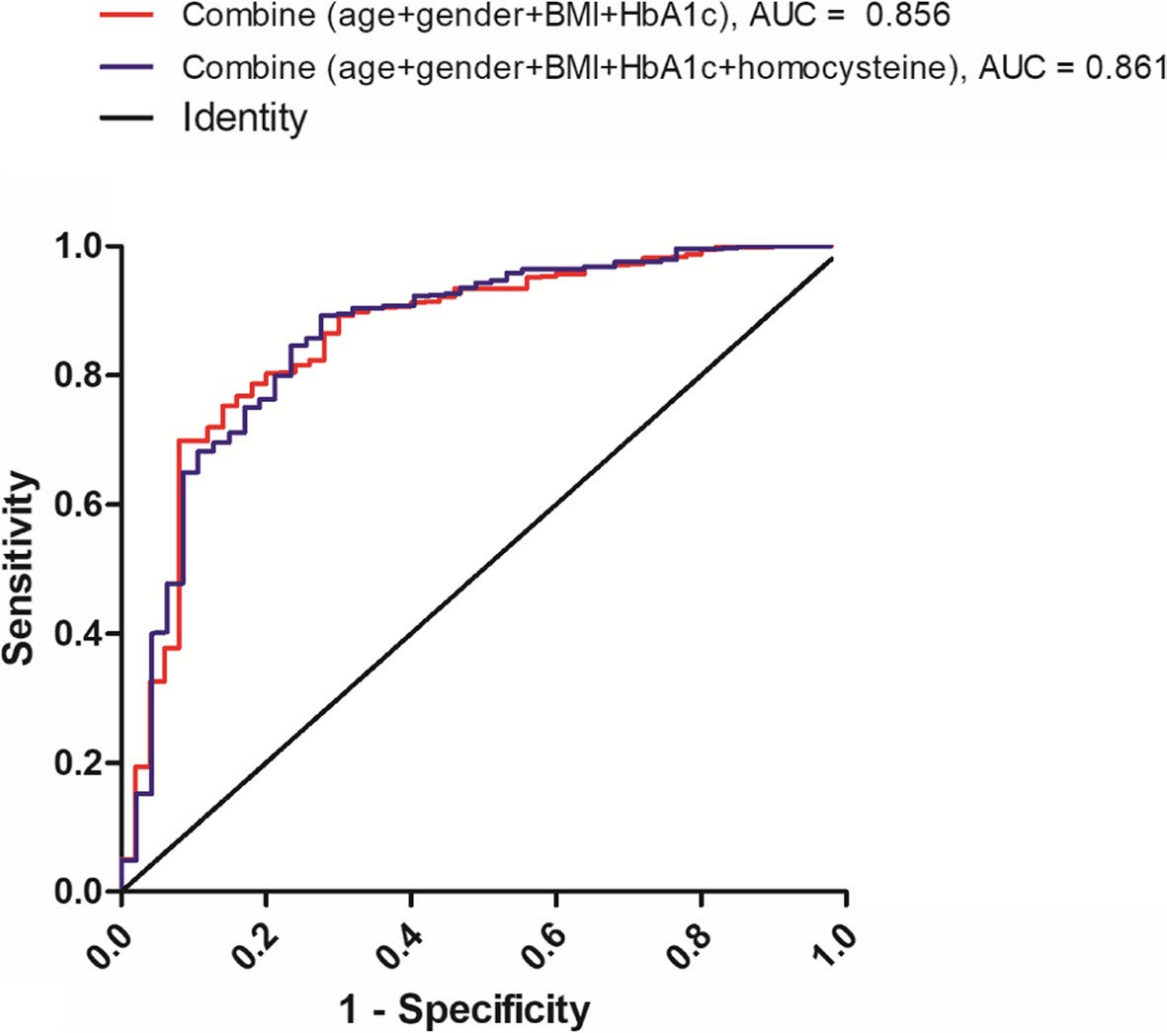
The ROC curve shows the abilities of various clinical indices to discriminate T2DM patients with or without sarcopenia

Since age, sex, BMI and HbA1c as well as homocysteine were all independent markers of sarcopenia, we utilized ROC curves to explore whether a combination of these variables could act as diagnostic markers to identify sarcopenia in older T2DM patients. We found that the combination of conventional indicators including age, sex, BMI and HbA1c, provided a reliable discrimination effect with an area under the curve (AUC) of 0.856. Importantly, when homocysteine was added to the model, the value of the ROC curve was further improved, with an AUC of 0.861 (Fig. 1).

## Discussion

Sarcopenia is a generalized, progressive muscle disease [1]. In the current study, we identified an 8.9% prevalence of sarcopenia in an older Chinese T2DM cohort. Additionally, we demonstrated a positive correlation of homocysteine (OR: 2.829; 95% CI, 1.064–7.525) and hs-CRP (OR: 1.021; 95% CI, 1.001–1.042) with the components of sarcopenia, and the relationship remained significant after adjustment for age, sex, BMI and HbA1c. Importantly, hs-CRP and homocysteine may serve as new biomarkers of sarcopenia and contribute to the development of sarcopenia.

Sarcopenia is an age-related disease with reduced muscle mass and function, and its prevalence has risen rapidly in recent years [2]. There is a significant variability about the prevalence of sarcopenia [3]. In older healthy people (≥ 60 years old), the prevalence of sarctaopenia varies from 5% to 50%, which has resulted in a significant reduction in the quality of life of elderly individuals [18]. The prevalence of sarcopenia in Europe is between 11% and 20% in healthy men and women aged over 60 years in 2016 [24]. Among Japanese residents (≥ 65 years old), the prevalence of sarcopenia is 11.5% in men and 16.7% in women [25]. Importantly, the prevalence of sarcopenia in elderly Japanese diabetes patients is even higher, about 18.7% [26]. A systematic review and meta-analysis from 20 studies including over 50,000 participants demonstrated that individuals with diabetes had greater likelihood of having sarcopenia [27]. A report from the Korean Sarcopenic Obesity Study, in which sarcopenia was defined using the skeletal muscle index only, found the prevalence of sarcopenia in patients with T2DM is much higher than the controls (15.7% vs. 6.9%) [12]. Similarly, Çeliker et al. [28] reported a higher prevalence of sarcopenia in T2DM individuals (21.4%) compared with those without T2DM (15.1%) in Turkey. In our study, we reported that the prevalence of sarcopenia in older adults with T2DM was 8.9% (mean 67.6 years). Our results were similar to the data from Mori H et al. in Japanese patients with T2DM (7.2%, with mean age of 63.2) [29]. However, our results were lower than 24% from Singapore diabetic patients with a mean age of 68.3 [30] and 28.5% from Malaysian in older T2DM patients [31]. The shortage of established diagnostic criteria for low SMI and the age deviation may explain these differences. In addition, studies have shown that the association between sarcopenia and T2DM varies with different adjustment methods of skeletal muscle mass [32]. Bahat et al. [33] reported that the association between muscle mass and functionality is better when skeletal muscle mass was adjusted by BMI rather than height^2^. Thus, the prevalence of sarcopenia in T2DM individuals may depend on different sarcopenia definitions, ethnicity, living settings as well as age, sex, BMI and comorbidities [34].

Additionally, hyperglycemia is extremely detrimental to muscle health and function and numerous studies have reported that patients with T2DM had a high risk of sarcopenia [12–14]. A longitudinal Korean study has reported that poor glycemic control (HbA1c above 8.5%) is harmful to muscle performance [35]. Also, we found that the patients with sarcopenia showed worse glucose control (9.2 ± 2.2 vs. 8.4 ± 1.8%, *P* = 0.008), which was confirmed by several other observations [36, 37], therefore, leading to the poor prognosis of diabetes. The hypothesized mechanism is the myosin glycation and mitochondrial dysfunction [37]. Clinically, screening T2DM patients who have sarcopenia and then giving targeted treatment and intervention may provide a more positive impact on the prognosis of both diseases.

Additionally, we found a negative correlation between the proinflammatory cytokines hs-CRP, IL-6, and SMI. In line with our results, recent studies of T2DM adults in Indonesians [38], the United Kingdom [39], and Korean [40] also showed an inverse correlation between SMI and hs-CRP. A large-scale meta-analysis of morbid participants also illustrated particularly higher concentrations of hs-CRP in subjects with sarcopenia. The relationships between IL-6 and sarcopenia differs in different studies. Grosicki et al. [41] and Visser et al. [20] reported the close correlation of low skeletal muscle mass with high serum levels of IL-6. The potential mechanism is the enhanced muscle catabolism caused by the chronic inflammation [10]. Importantly, protein synthesis in skeletal muscle demonstrates an inverse association with CRP and IL-6 [42]. However, Bano et al. [43] reported that patients with sarcopenia experienced significantly higher levels of CRP, whilst no significant differences emerged for IL-6, which is consistent with our study. It has previously been shown that the sole action of IL-6 is not enough to induce muscle wasting, the catabolic effect of IL-6 is dependent on the synergistic interaction with other factors mediating the inflammatory response. The correlation between interleukin-6 and sarcopenia remains to be further explored. A series of studies have revealed the causal relationship between inflammatory markers and decreased muscle mass [20, 44]. In a study of rats, it was found that the infusion of inflammatory factors can result in the atrophy of skeletal muscle [45]. To summarize, chronic inflammation plays a vital role in the progression of sarcopenia.

As a sulphur-containing amino acid, homocysteine is an important intermediate in the metabolism of methionine and cysteine [46]. The increased serum homocysteine contributes to tissue damage by oxidative stress, thus resulting in age-correlated diseases, notably cardiovascular disease, bone and glucose metabolism, and muscle function [21]. Herein, we found that serum homocysteine served as a positive predictor of sarcopenia, and the correlation remained even after adjustment for HbA1c. The results coincide with those from Lee et al., who observed the positive correlation of sarcopenia and homocysteine in healthy older residents [47]. Moreover, an early study with a large sample size from the United States also found an inverse association between homocysteine and gait speed, and quadriceps strength [48]. The mechanism of hyperhomocysteinemia and sarcopenia is still unclear. The general perception is that the hyperhomocysteinemia may mediate the imbalance of enzymatic and non-enzymatic cross-linking of collagen [48]. Veeranki et al. [49] established a rodent model to explore the pathophysiological mechanisms of homocysteine and sarcopenia and suggested that the impaired muscle fiber regeneration and energy production disorders caused by mitochondrial dysfunction were the reasons for the development of sarcopenia. Recently, a potential crosstalk between renin-angiotensin system (RAS) and homocysteine has been suggested [50]. It has been reported that the RAS was involved in the development of insulin resistance in skeletal muscle and the increase of angiotensin II (Ang II) levels was approved to induce skeletal fiber wasting through enhanced protein degradation and apoptosis as well as decreased protein synthesis [51–53]. However, although homocysteine is still an independent risk factor for sarcopenia in our study after adjusting for several confounders, adding homocysteine to the ROC improves the fit slightly. Thus, we speculate that homocysteine may play a minor role in diagnosing sarcopenia. Further basic and clinical studies should be carried out to reveal the mechanisms underlying sarcopenia and high serum levels of homocysteinemia considering the quickly increasing prevalence of sarcopenia, and it is ineffective treatment.

Growing attention has been given to the advantage of reversing sarcopenia by reducing the level of inflammatory markers and homocysteine. In a model of aged rats, Rieu et al. [54] discovered that the muscle protein synthesis was restored by non-steroidal anti-inflammatory drugs (NSAIDs) which was used to suppress low-level inflammation. Furthermore, the beneficial effect of NSAIDs in muscle dysfunction was also confirmed in older people [55]. For homocysteine, it is well known that the supplementation with vitamin B12 and folic acid can correct hyperhomocysteinemia to reverse its adverse effects [56]. A previous nutrition intervention study from Japan demonstrated that vitamin B12 and folic acid could reduce the probability of fracture incidence [57], thus improving the strength and function of muscle [58]. However, a large review on the intervention of vitamin B12 to reduce the concentrations of homocysteine showed no differences in adverse outcomes [57]. Since there is not enough information about the therapeutic effect of these medicines, further in-depth research is particularly warranted. In addition, when we compared the sarcopenia group to the non-sarcopenic group, patients with sarcopenia showed a higher proportion of male subjects and had significantly lower mean BMI values. Our outcomes were consistent with several other observational studies [59–62]. Hormone differences may be the main reason for these outcomes. Although this study adds much information to the field of sarcopenia, there are still several limitations involved in the present study. First, this study was a cross-sectional study, and causal relationships therefore could not be determined. Second, our results come from Chinese adults with T2DM, which may not be applicable to other ethnic groups without replication. Thirdly, the information about drugs patients were taking for diabetes and the parameters such as creatine phosphokinase (CPK) and vitamin B12 is missing in our study, which may cause bias. Fourthly, we included the most common inflammatory markers such as hs-CRP and IL-6, although many others (TNF-α, IL-10, etc.) that have been implicated in sarcopenia. Finally, the exclusion criterion was designed to exclude patients who have neurological disease or sequelae of cerebrovascular disease. The criterion may lead to the omission of some patients with sarcopenia, and ultimately cause the deviation of the results. Besides, according to the recommendation from EWGSOP2, SARC-F questionnaire stands as one of the best tools to evaluate sarcopenia [63–65]. Without screening for SARC-F in this study is of course a lacking, we will definitely consider validation of SARC-F in our future research.

Nonetheless, this study enriches our knowledge of the prevalence of sarcopenia in older Chinese adults with T2DM, and reveals the relationship and intrinsic connection of inflammatory markers and homocysteine with sarcopenia and its components.

## Conclusions

The current study investigated the prevalence of the sarcopenia in older Chinese adults with T2DM, and found that the concentrations of hs-CRP and homocysteine were independent predictors of sarcopenia. These indicators may play an important role in the pathological process and development of sarcopenia.

## Data Availability

The datasets used and analyzed during the current study are available from the corresponding authors for reasonable request.

## Abbreviations

T2DM: Type 2 diabetes mellitus
EWGSOP: European working group on sarcopenia in older people
ROC: Receiver operating characteristic
CI: Confidence interval
AUC: Area under the curve
OR: Odds ratio
Hs-CRP: High-sensitive C-reactive protein
DXA: Dual-energy x-ray absorptiometry
SPSS: Statistical package for social science
BMI: Body mass index
LDL-C: Low density lipoprotein cholesterol
HDL-C: High-density lipoprotein cholesterol
HbA1c: Hemoglobin A1c/glycated hemoglobin
ASM: Appendicular skeletal muscle
SMI: Height-adjusted appendicular skeletal muscle mass
WBC: White blood cell
RBC: Red blood cell
PLT: Blood platelet
HGB: Hemoglobin
SD: Standard deviation
RAS: Renin-angiotensin system
Ang II: Angiotensin II
NSAIDs: Non-steroidal anti-inflammatory drugs
CPK: Creatine phosphokinase
